# Hormonal Regulation of Ovarian Bursa Fluid in Mice and Involvement of Aquaporins

**DOI:** 10.1371/journal.pone.0063823

**Published:** 2013-05-22

**Authors:** He Zhang, Ying Zhang, Huashan Zhao, Yunfang Zhang, Qi Chen, Hongying Peng, Li Lei, Jingqiao Qiao, Junchao Shi, Zhonghong Cao, Enkui Duan, Yaping Jin

**Affiliations:** 1 College of Veterinary Medicine, Northwest A & F University, Yangling, Shaanxi, China; 2 State Key Laboratory of Reproductive Biology, Institute of Zoology, Chinese Academy of Sciences, Beijing, China; 3 University of Chinese Academy of Sciences, Beijing, China; University of Santiago de Compostela School of Medicine - CIMUS, Spain

## Abstract

In rodent species, the ovary and the end of oviduct are encapsulated by a thin membrane called ovarian bursa. The biological functions of ovarian bursa remain unexplored despite its structural arrangement in facilitating oocytes transport into oviduct. In the present study, we observed a rapid fluid accumulation and reabsorption within the ovarian bursa after ovarian stimulation (PMSG-primed hCG injection), suggesting that the ovarian bursa might play an active role in regulating local fluid homeostasis around the timing of ovulation. We hypothesized that the aquaporin proteins, which are specialized channels for water transport, might be involved in this process. By screening the expression of aquaporin family members (*Aqp1-9*) in the ovarian tissue and isolated ovarian bursa (0, 1, 2 and 5 h after hCG injection), we found that AQP2 and AQP5 mRNA showed dynamic changes after hCG treatment, showing upregulation at 1–2 h followed by gradually decrease at 5 h, which is closely related with the intra-bursa fluid dynamics. Further immunofluorescence examinations of AQP2 and AQP5 in the ovarian bursa revealed that AQP2 is specifically localized in the outer layer (peritoneal side) while AQP5 localized in the inner layer (ovarian side) of the bursa, such cell type specific and spatial-temporal expressions of AQP2 and 5 support our hypothesis that they might be involved in efficient water transport through ovarian bursa under ovulation related hormonal regulation. The physiological significance of aquaporin-mediated water transport in the context of ovarian bursa still awaits further clarification.

## Introduction

The ovary is an important organ for oocyte formation and release. In rodent species, the ovary is encapsulated by a thin membrane structure that fused with the end of the oviduct, which is called the ovarian bursa. The ovarian bursa shields the ovary from the peritoneal environment and provides a fluid chamber for oocytes development and ovarian function. Upon ovulation, the oocytes are expulsed into the ovarian bursa along with ovarian fluids, the structure of ovarian bursa is supposed to facilitate the retrieval and transport of ovulated oocytes into oviduct, and this idea has been reinforced by the observation that in rats after surgical bursa removal, the oocytes could not normally enter the oviduct [1].

In murine species, the ovarian bursa consists of three major layers, an interior layer facing the ovarian epithelium, an exterior layer facing the peritoneal cavity and a central layer of connective tissue [2]. It has been demonstrated in murine species, the formation of ovarian bursa began after the postnatal day 9 and its complete formation progressed gradually after first ovulation [3]. The clear structural arrangement of ovarian bursa and its close relationship with ovary have been utilized for cell/tissue transplantation, drug/reagent delivery in ovarian functional studies, which have been proven to be successful [4], [5], [6], [7]. However, the physiological function of ovarian bursa remains poorly understood. An empirical idea is that the ovarian bursa seems to be closely related to fluid homeostasis and substance transport around ovary, thus providing optimal environments for its function [2]. It has long been noticed that just before ovulation, there are dynamic fluid changes within the ovarian bursa [8], the accumulation and drainage of fluid have been previously implicated to link with the bursa lymphatic system [9], [10], [11], [12], however, the molecular basis for such rapid fluid transport has not been clarified.

In present investigation, we found a transient intra-bursa fluid accumulation and reabsorption within the first 5 hours after PMSG (pregnant mare's serum gonadotropin)-primed hCG (human chorionic gonadotrophin) administration. We hypothesized that the rapid fluid regulation within ovarian bursa might be related to the aquaporin family proteins, which are specialized channels for water permeability and showed a wide range of physiological functions [13], [14]. To date, at least nine aquaporin isoforms (AQP1-AQP9) have been confirmed to be expressed in the male and female reproductive tract. Their specific expression pattern together with their regulation by steroid sex hormones provide indirect evidences of a role for AQPs in reproductive physiology [15], [16], while the expression of aquaporins in ovarian bursa have not been studied. Combining time-interval measurements of intra-bursa fluid volume after ovarian stimulation (0, 1, 2, 5 h after PMSG-primed hCG injection) and RT-PCR (Reverse Transcription-Polymerase Chain Reaction) screening of the aquaporin family members at each time point, we discovered that the rapid intra-bursa fluid accumulation and drainage are closely related to dynamic expressional changes of AQP2 and AQP5, which are localized at distinct layers of the ovarian bursa, suggesting coordinated roles in keeping local fluid environments. These data provided novel evidences suggesting that dynamic water channels expression under ovulation hormones is actively involved in bursa fluid homeostasis.

## Materials and Methods

### Ethics Statement

The Guidelines for the Care and Use of Animals in Research were followed. Mice care and handling were conducted in accordance with the Animal Research Committee guidelines of the Institute of Zoology, Chinese Academy of Sciences. The institute does not issue a number to each animal study, but there is an ethical committee to guide animal use. The contents in present study regarding animal uses were approved by the Animal Research Committee of the Institute of Zoology, Chinese Academy of Sciences.

### Animals

CD1 female mice (7–8 weeks) used in this study were purchased from Vital River Laboratories Co. Ltd. All mice were fed in the animal facility of Institute of Zoology, Chinese Academy of Sciences. The mice were maintained in 12 h light, 12 h dark conditions and given water and food freely. Ovarian stimulation was induced in 7–8 weeks old female mice by administration (ip) of 10 IU PMSG, 48 h later followed by either 10 IU hCG or the same volume of saline as control. Mouse were sacrificed at different time points after 0 h, 1 h, 2 h and 5 h hCG injection, the ovaries (including the encapsulating bursa) were flash frozen in liquid nitrogen for RNA extraction and frozen sections. Some ovaries (including the encapsulating bursa) were further processed for mechanical isolation of ovary and bursa. The isolated ovaries and bursas were used for RNA extraction.

### RNA Extraction, RT-PCR and Real-time PCR

Total RNA was extracted from fresh tissues using the TRIzol reagent (Invitrogen), and genomic DNA was removed using the RNase-free DNase (Promega) as previously described [17]. After reverse transcription, RT-PCR was conducted for *Aqp1-9* (30 cycles) and *Beta-actin* (23 cycles). The positive control consists of a mixture of cDNAs from lung, bladder, liver, and kidney, which covers the expression of all aquaporin family members, water served as the negative control. The primers used for amplifying mouse *Aqp1-9* and *β-actin*: *Aqp1*, forward: 5′-TGCGTTCTGGCCACCACTGAC-3′, reverse: 5′-GATGTCGTCAGCATCCAGGTC-3′; *Aqp2*, forward: 5′-GCCATCCTCCATGAGATTACC-3′, reverse: 5′-ACCCAGTGATCATCAAACTTG-3′; *Aqp3*, forward: 5′-CTGGACGCTTTCACTGTGGGC-3′, reverse: 5′-GATCTGCTCCTTGTGTTTCATG-3′; *Aqp4*, forward: 5′-CTGGAGCCAGCATGAATCCAG-3′, reverse: 5′-TTCTTCTCTTCTCCACGGTCA-3′; *Aqp5*, forward: 5′-CTCTGCATCTTCTCCTCCACG-3′, reverse: 5′-TCCTCTCTATGATCTTCCCAG-3′; *Aqp6*, forward: 5′-TCTGTTCTGCCCTGGCCTGTG-3′, reverse: 5′-ACCGCCTGGCCAGTTGATGTG-3′; *Aqp7*, forward: 5′-GAGTCGCTAGGCATGAACTCC-3′, reverse: 5′-AGAGGCACAGAGCCACTTATG-3′; *Aqp8*, forward: 5′-GGGGCAGCCTTTGCCATCGT-3′, reverse: 5′-AAGAGGCCAGCCAGGAGGGG-3′; *Aqp9*, forward: 5′-CCTTCTGAGAAGGACCGAGCC-3′, reverse: 5′-CTTGAACCACTCCATCCTTCC-3′; *β-actin*, forward: 5′-TGGAATCCTGTGGCATCCATGAAAC-3′, reverse: 5′-TAAAACGCAGCTCAGTAACAGTCCG-3′.

For *Aqp2* and *Aqp5*, SYBR Green-based real-time PCR was carried out using LightCycler 480 II (Roche) as pre-incubation at 95°C for 2 min; followed by amplification at 95°C for 15 sec, 55°C for 15 sec, 68°C for 15 sec, 40 cycle; the program for the melting curve analysis was 95°C for 5 sec, 65°C for 1 min. Primers for real-time PCR: *Aqp2*, forward: 5′-TCATCGGTTCCCTCCTCTAC-3′, reverse: 5′-CGTTCCTCCCAGTCAGTGTCC-3′; *Aqp*5, forward: 5′-CTCTGCATCTTCTCCTCCACG-3′, reverse: 5′-TCCTCTCTATGATCTTCCCAG-3′; *Gapdh*, forward: 5′-GGTTGTCTCCTGCGACTTCAACAGC-3′, reverse: 5′-CGAGTTGGGATAGGGCCTCTCTTGC-3′.

### Immunofluorescence Examination

Immunofluorescence examinations were performed as previously described [17]. Frozen sections (10 µm) were fixed in 4% paraformaldehyde (PFA) for 10 min at room temperature, then 0.5% Triton treated for 5 min at room temperature after washing with PBS 3 times. Nonspecific binding was blocked in PBS with 5% bovine serum albumin (BSA) for 1 h at 37°C followed by incubation with rabbit anti-AQP2 (sc-28629, Santa Cruz Biotechnology) and rabbit anti-AQP5 (raised in MBL, against C-terminal of mouse AQP5∶230–244 CLSLHDRVAVVKGTYE). Primary antibody diluted in blocking solution overnight at 4°C in a humid chamber. After three washes in PBS, the sections were incubated with secondary antibody (Goat anti Rabbit IgG/FITC; ZF-0311, Beijing Zhong Shan Golden Bridge Biological Technology CO, LTD) diluted in blocking solution for 1 h at 37°C. After wash three times in PBS. Nuclei were stained with 5 µg/mL of Propidium Iodide (Sigma) for 10 minutes. Slides were photographed under an immunofluorescence microscope (Nikon 80i).

### Measurements of Intra-bursa Fluid Volume

To measure the volume of intra-bursa fluid, we have developed a method by releasing the intra-bursa fluid (ruptured by a clean needle) on to a filter paper to form a circled stain area, and then obtained the fluid volume by comparing the stain sizes with standardized fluid volume drops on the same kind of filter paper. This method allowed us to measure the intra-bursa fluid volume with acceptable precision and repeatable results.

### Statistical Analysis

Statistical analyses were performed with SPSS 17.0. All data are present as mean ± S.E.M of at least three independent experiments. Results were analyzed by one-way ANOVA or independent *t* test. *P*<0.05 was considered to be statistically significant.

## Results

### Ovarian Bursa Fluid Showed Dynamic Volume Changes after PMSG-primed hCG Injection

**Figure 1:**
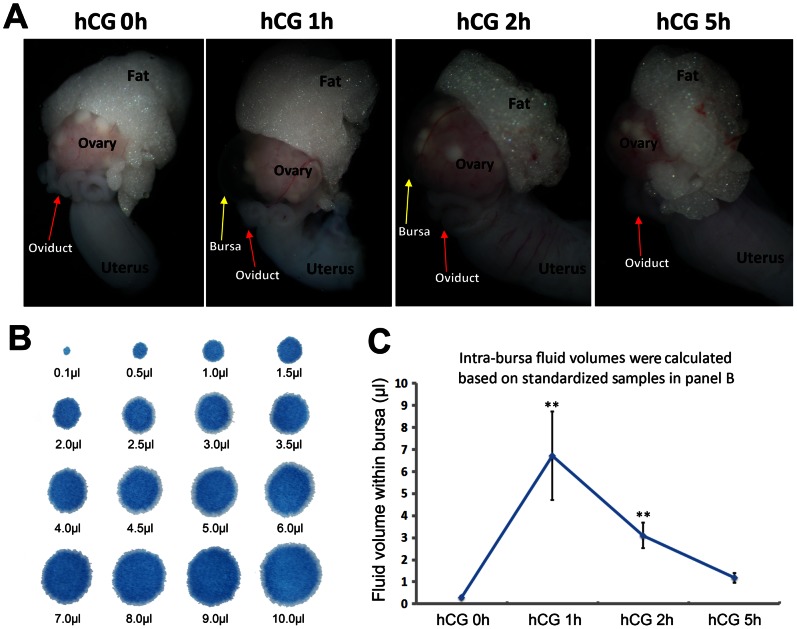
Intra-bursa fluid changed dynamically after PMSG-primed hCG injection. (**A**) Demonstrative photos showing rapid accumulation and reabsorption of intra-bursa fluid. (**B**) Standardized fluid-drop stains on a filter paper with defined volumes. (**C**) Intra-bursa fluid volumes at different time intervals after PMSG-primed hCG injection. For each time point, n = 10. Error bars represent S.E.M., **P<0.01.

During our standard protocols for mouse superovulation, we have constantly noticed that after the PMSG treatment for 48 h, a single hCG injection induced dynamic fluid changes within the ovarian bursa. In present study, we looked into this phenomenon and carefully examined the temporal order of the fluid dynamics by measuring the intra-bursa fluid volumes at 0, 1, 2, and 5 hours after PMSG-primed hCG injection. As shown by the illustrative pictures (Fig. 1A), there is a rapid fluid accumulation 1 h after hCG, followed by gradual reabsorption 2–5 h after hCG injection. By further measure the volume of intra-bursa fluid according to standardized fluid volume drops (Fig. 1B), we found that the statistic data (Fig. 1C) were consistent with our gross observations as in Figure 1A, highlighting a rapid intra-bursa fluid dynamics triggered by ovulation related hormones.

### AQP2 and AQP5 mRNA Showed Temporal Changes Correlating with the Intra-bursa Fluid Dynamics after PMSG-primed hCG Injection

**Figure 2:**
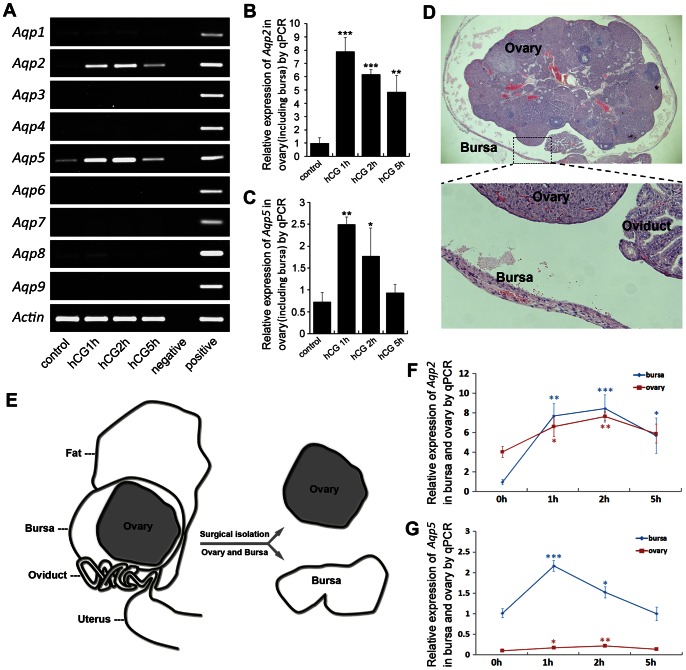
AQP2 and AQP5 transcripts showed temporal change correlating with the intra-bursa fluid dynamics. (**A**) Expression profiles of aquaporin family members (*Aqp1-9*) after PMSG-primed hCG injection reveal dynamic changes of *Aqp2* and *Aqp5* in the ovary (containing encapsulating bursa). (**B, C**) Quantitative RT-PCR examinations of *Aqp2* (**B**) and *Aqp5* (**C**) expression. n = 3 for each time point. Error bars represent S.E.M., *P<0.05, **P<0.01, ***P<0.001. (**D**) Demonstrative photos showing the structure of ovary and bursa. (**E**) Illustrative pictures showing the surgical isolation of ovary and bursa, the isolated ovary and bursa were processed for RNA extraction respectively. (**F, G**) Real-time PCR examinations showed relative changes of *Aqp2* (**F**) and *Aqp5* (**G**) in isolated ovary and bursa at different time points after hCG injection. Relative expression levels for *Aqp2* and *Aqp5* were normalized between ovary and bursa. Statistics were made to compare the expression levels of different time points to 0 h for each tissue. *P<0.05, **P<0.01, ***P<0.001. n = 3 or 4 for each time point. Error bars represent S.E.M.

The rapid fluid turnover within ovarian-bursa compartment had led us to hypothesize that the aquaporin family, which is specialized for rapid trans-membrane water transport, might play an active role in this process. Therefore, we first collected the ovary (with the bursa encapsulating it) at different time points after PMSG-primed hCG injection, and screened the expression profiles of different aquaporin family members *Aqp1-9* by RT-PCR. As shown in Figure 2A, we found that among the aquaporin family, *Aqp2* and *5* are sensitively regulated by the hormonal treatments and their expression levels showed dynamic changes closely correlated with the intra-bursa fluid volume as described in Figure 1A. The transcriptional level of AQP2 and AQP5 were further confirmed by performing real-time PCR at corresponding time points (Fig. 2B,C), suggesting that they might be actively involved in the process of rapid bursa fluid regulation.

### *Aqp2* and *Aqp5* are Separately Regulated in the Bursa and the Ovary

As the ovary and bursa are two distinct tissue compartments (Fig. 2D), we tried to further examine whether the dynamic changes of *Aqp2* and *Aqp5* mainly originated from the ovary or the bursa, or both. As shown in Figure 2E, we mechanically peeled the bursa off the ovary, and collected each part of them for further real-time PCR examination of *Aqp2* and *5*. The results indicated that the *Aqp2* showed more dynamic changes in the bursa than the ovarian tissue (Fig. 2F), while the expression level of *Aqp5* is much lower in the ovarian tissue than in the bursa (Fig. 2G).

### AQP2 and AQP5 are Region Specifically Localized in Ovarian Bursa and Ovary

**Figure 3:**
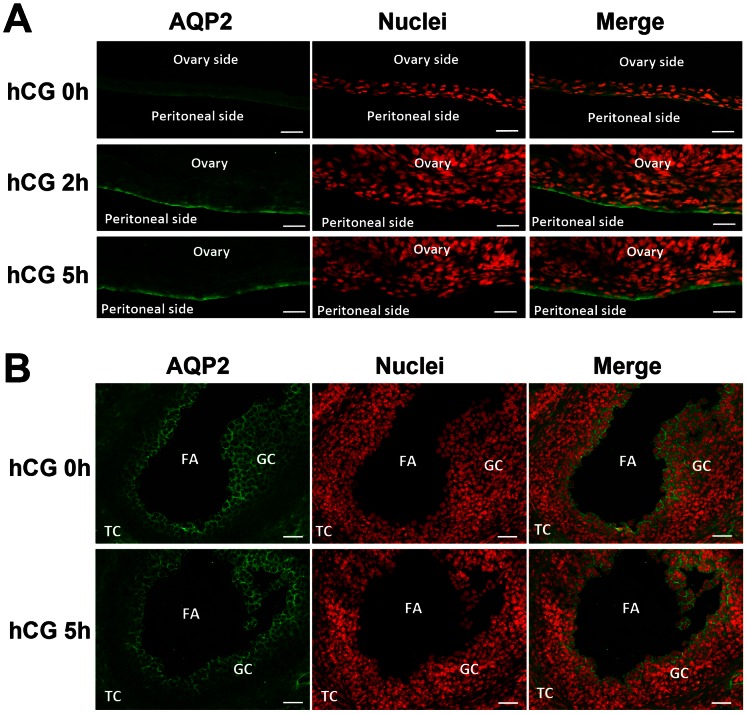
AQP2 protein localization in ovary and ovarian bursa after PMSG-primed hCG injection. (**A, B**) Immunofluorescence assay for AQP2 in ovarian bursa (**A**) and ovary (**B**). FITC-labeled AQP2 antibody is in green, and propidium iodide-labeled nuclei are in red. FA: follicular antrum. GC: granulosa cell. TC: theca cells. Scale bars: 50 µm.

**Figure 4:**
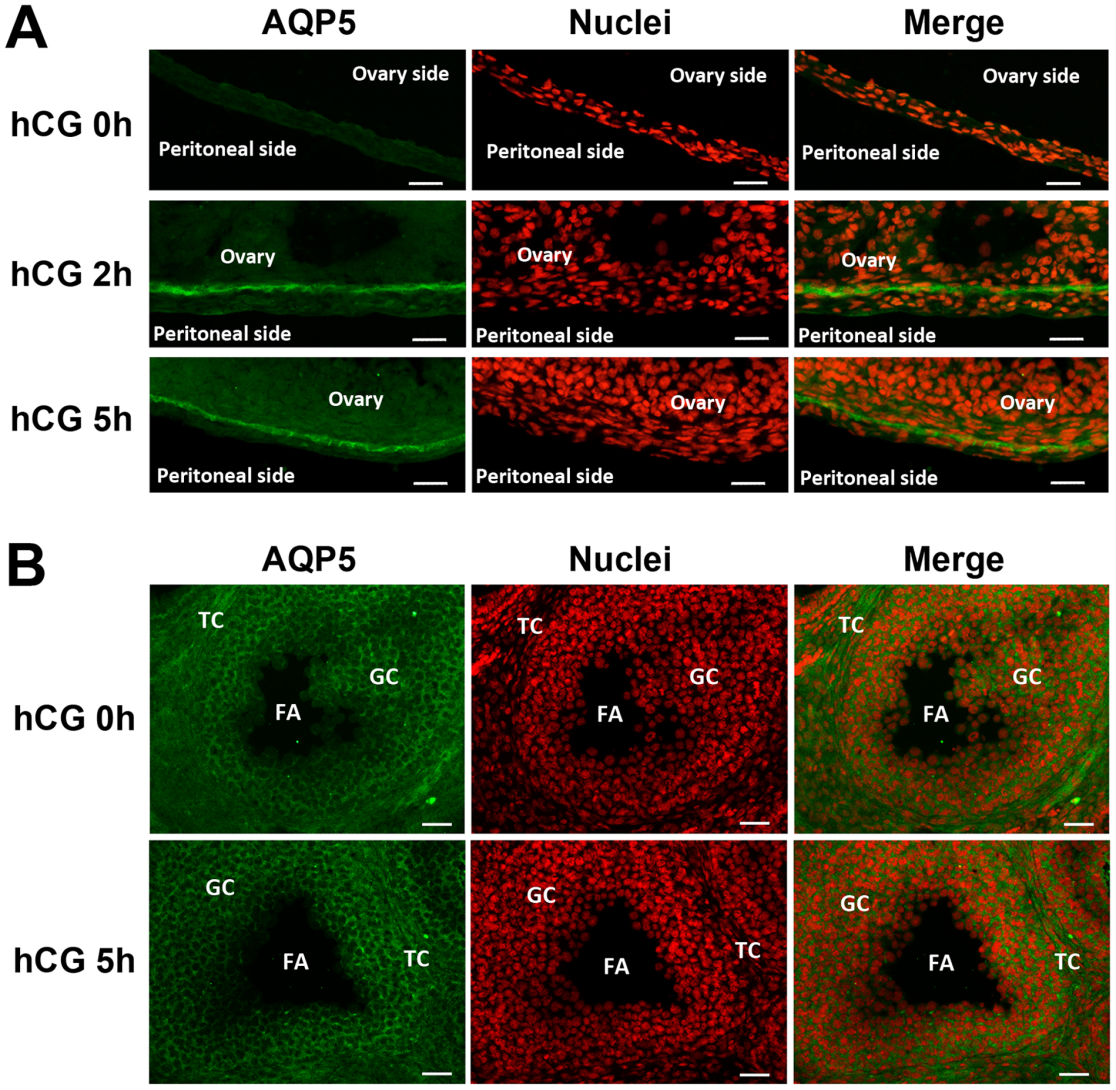
AQP5 protein localization in ovary and ovarian bursa after PMSG-primed hCG injection. (**A, B**) Immunofluorescence assay for AQP5 in ovarian bursa (**A**) and ovary (**B**). FITC-labeled AQP5 antibody is in green, and propidium iodide-labeled nuclei are in red. FA: follicular antrum. GC: granulosa cell. TC: theca cells. Scale bars: 50 µm.

To identify the protein localization of AQP2 and AQP5 in ovary and bursa, we further performed immunofluorescence assay on frozen tissue sections. As shown in Figure 3 and Figure 4. The immunofluorescence staining showed that the protein levels of AQP2 and AQP5 are more dynamically expressed in the bursa, while the intensities in the ovary were more constant. Particularly, it is very interesting to find that at 2 h and 5 h after hCG, AQP2 intensely localized at the outer layer of the bursa (Fig. 3A), while AQP5 localized at the inner layer of the bursa (Fig. 4A). Such coordinated arrangements of AQP2 and AQP5 at distinct sides of ovarian bursa suggested that they might play collaborative roles for trans-bursa fluid transportation, possibly responsible for different physiological roles such as fluid “in” and “out”. Also, it is very interesting to notice that within the ovary, the AQP2 and AQP5 are localized in complementary region/cell populations in the granulosa and theca cells, suggesting their distinct and collaborative roles in intra-ovarian fluid homeostasis.

## Discussion

In this study, we discovered that in adult mouse, pre-ovulation hormonal stimulation (PMSG-primed hCG injection) induced a rapid fluid accumulation and reabsorption within the ovarian bursa, which is closely associated with the spatial-temporal expressions of two aquaporin proteins AQP2 and AQP5, showing dynamic up and down regulations. At the protein level, AQP2 localized on the peritoneal side while AQP5 on the ovarian epithelial side of ovarian bursa, such interesting arrangements of AQP2 and AQP5 on the distinct compartments suggested their coordinated roles in balancing intra-bursa fluid homeostasis.

The ovarian bursa usually contains small amount of fluid except for the substantial increase at the time near ovulation. It has been suggested that the lymphatic stomata within the ovarian bursa might took part in the bursa fluid/substance circulation from the ovarian cavity to the vascular system, mainly due to its closely related structure [2], and its regulation by steroid hormones [18]. The murine bursa fluid had been described to increase 10 h after hCG administration right before ovulation [8], and the origin of fluid at this pre-ovulation period was suggested to derive partly from the plasma in the follicle walls and partly from the follicular fluid of ovulating oocytes [19]. Such increased bursa fluid was supposed to lubricate the route by which the ovulated oocytes would pass through later on.

In our present study, we found an even earlier and transient intra-bursa fluid accumulation and reabsorption within the first 5 hours after PMSG-primed hCG administration, which seems to be more tightly regulated by hormonal regulation and the coordinated expression of specialized water channel AQP2 and 5. Both AQP2 and AQP5 belong to the classic members of aquaporin family that solely permeable to water. AQP2 is abundantly localized in the principal cells of the kidney, which is critical for the vasopressin-dependent urine concentration [20]. AQP5 has been firstly demonstrated to be actively involved in fluid secretion in secretory glands such as salivary glands, submucosal glands et.al, and its deletion resulted in decreased fluid secretion [21], [22]. Recently, more evidences have showed that AQP2 and AQP5 expression could be activated by estrogen, and estrogen response elements have been identified in both *Aqp2* and *Aqp5* promoter regions [23], [24]. In present study, it is possible that the transient up-regulation of AQP2 and AQP5 in the ovarian bursa could be due to the estrogen peak after PMSG-hCG treatment. Also, the peri-ovarian adipose tissue might be involved in the regulation of AQP expression in the ovarian bursa as well. For example, the adipocyte-derived hormone leptin, has been shown to regulate reproductive function by altering the sensitivity of the pituitary gland to gonadotropin-releasing hormone (GnRH) and acts at the ovary to regulate follicular and luteal steroidogenesis [25]. Moreover, leptin has been shown to regulate expression of several AQPs in the adipose tissue [26], and might also be involved in the regulation of ovarian/bursa AQPs. At present time, the origin and the outlet of the intra-bursa fluid in the first hours after hCG administration have not been clarified, given the specialized expression of AQP2 and AQP5 at the inner and outer layers of ovarian bursa, one possibility is that the intra-bursa fluid homeostasis at this period might be directly linking with the peritoneal fluid environments.

So far, several aquaporin knockout mice have shown reproductive phenotypes in both male and female. For example, in males, *Aqp3* knockout mice showed defects in sperm osmoadaptation thus impaired male fertility [27]. In females, *Aqp4* knockout mice showed decreased female fertility [28], while *Aqp8* knockout mice showed increased fertility [29], [30], both related to changed ovarian function, but the detailed mechanisms are not understood. Previous reports have revealed that several aquaporin members are expressed in the different compartments of ovary including follicles, oocytes, granulosa cells, theca cells and ovarian epithelium in diverse species [29], [31], [32], [33], but there have not been reports regarding the aquaporin expression in the ovarian bursa. In this regards, our data provide the first evidence showing clear and dynamic expression of AQP2 and AQP5 in the mouse ovarian bursa in a physiologically related and hormonally regulated manner, suggesting active roles in regulating intra-bursa fluid homeostasis. While the mutually complementary expression of AQP2 and AQP5 in the granulosa and theca cells suggesting their role in follicular fluid formation and regulation as previously discussed [31]. However, despite these expressional clues, it should be noticed that *Aqp5* knockout mice have not been reported to have related reproductive phenotypes [16], and *Aqp2* knockout mice die early before they reach puberty [34]. Therefore, the detailed physiological functions of AQP2 and AQP5 in the ovarian bursa still await future clarifications.

In conclusion, the present study revealed dynamic intra-bursa fluid changes after PMSG-primed hCG administration, as well as spatial-temporal associated expression of AQP2 and AQP5 in the distinct compartments of ovarian bursa. Our data suggested that expression of water channels under ovulation hormones is actively involved in bursa fluid homeostasis.
